# A Case of Disappearing Ascites: Late Presentation of Acute Portal Vein Thrombosis After a Motor Vehicle Accident

**DOI:** 10.7759/cureus.45926

**Published:** 2023-09-25

**Authors:** Teresa Del Rio, Dayana Reveron, Susan Ramdhaney

**Affiliations:** 1 Internal Medicine and Hepatology, Virginia Mason Medical Center, Seattle, USA; 2 Internal Medicine, Wyckoff Heights Medical Center, Brooklyn, USA; 3 Gastroenterology, Wyckoff Heights Medical Center, Brooklyn, USA

**Keywords:** motor vehicle accident, liver function, hepatology, gross ascites, portal vein tumor thrombosis

## Abstract

Portal vein thrombosis (PVT) has been usually diagnosed as a complication secondary to cardiac, hepatic, and malignant etiologies, but it has rarely been described in the setting of blunt abdominal trauma. This case depicts an older male who presented to the emergency department with progressive ascites and lower extremity edema within two weeks after a motor vehicle accident (MVA). Ascitic fluid analysis indicated the presence of portal hypertension, which prompted extensive evaluation to determine the etiology. Further workup, including hypercoagulable and malignancy screening, unveiled the diagnosis of acute PVT secondary to abdominal blunt force trauma, showing a rather rare presentation.

## Introduction

Portal vein thrombosis (PVT) is the narrowing or total occlusion of the portal vein secondary to a thrombus and accounts for approximately 5-20% of the cases of portal hypertension [1,2]. Common presentations of this phenomenon are divided into acute and chronic stages of disease. Acute stages of PVT present usually without symptoms or vague abdominal pain, fever, or chills, given compensatory mechanisms such as arterial vasodilation and collateral vessel development as early as a few days following the obstruction [1,2]. Meanwhile, chronic stages of PVT present with variceal bleeding, thrombocytopenia, or symptomatic splenomegaly [2]. Common etiologies of PVT are divided into inherited and acquired classifications. Inherited causes include procoagulant states such as factor V Leiden, prothrombin gene mutation, protein C deficiency, protein S deficiency, antithrombin deficiency, and increased factor VIII levels. However, the majority of PVT cases are due to acquired causes associated with primary or metastatic liver malignancy, advanced liver cirrhosis, inflammatory intra-abdominal disease, and myeloproliferative disease, with an estimated prevalence of up to 67%, 28%, 10%, and 3%, respectively [1,2]. Rare cases of PVT have been reported following abdominal blunt force trauma, both in isolated cases and organ injuries with a high incidence of morbidity and mortality [3]. Its pathophysiologic mechanism remains unclear, but a relationship between both endothelial damage and release of thrombus from smaller vessels has been proposed [4].

This case portrays an acute portal venous thrombosis as a rare complication following blunt abdominal trauma. We present a case of a patient with new-onset ascites and abdominal pain associated with PVT two weeks after a motor vehicle accident (MVA).

## Case presentation

A 62-year-old male with hypertension, diabetes mellitus type II, and chronic kidney disease presented to the emergency department with sharp right upper quadrant (RUQ) abdominal pain radiating to the right lower quadrant associated with bilateral lower extremity edema. Symptoms started two weeks prior to an MVA and had progressed since onset. The patient was hemodynamically stable and a febrile. Physical examination was remarkable for mild RUQ abdominal tenderness, distended abdomen with fluid wave present, and grade IV bilateral lower extremity edema. Remarkable laboratories are demonstrated in Table 1.

**Table 1:** Laboratory values AST, aspartate aminotransferase; ALT, alanine aminotransferase

Laboratory values		Results	Normal values	
Alkaline phosphatase		324 U/L	100-200 U/L	
AST		76 U/L	10-40 U/L	
ALT		128 U/L	10-55 U/L	
Albumin		2.9 g/dL	3.1-4.3 g/dL	
Total protein		6.2 g/dL	6.0-7.8 g/dL	
BUN		90 mg/dL	8-20 mg/dL	
Creatinine		5.48 mg/dL	0.7-1.3 mg/dL	
Hemoglobin		9.2 g/dL	14-17 g/dL	

**Figure 1:**
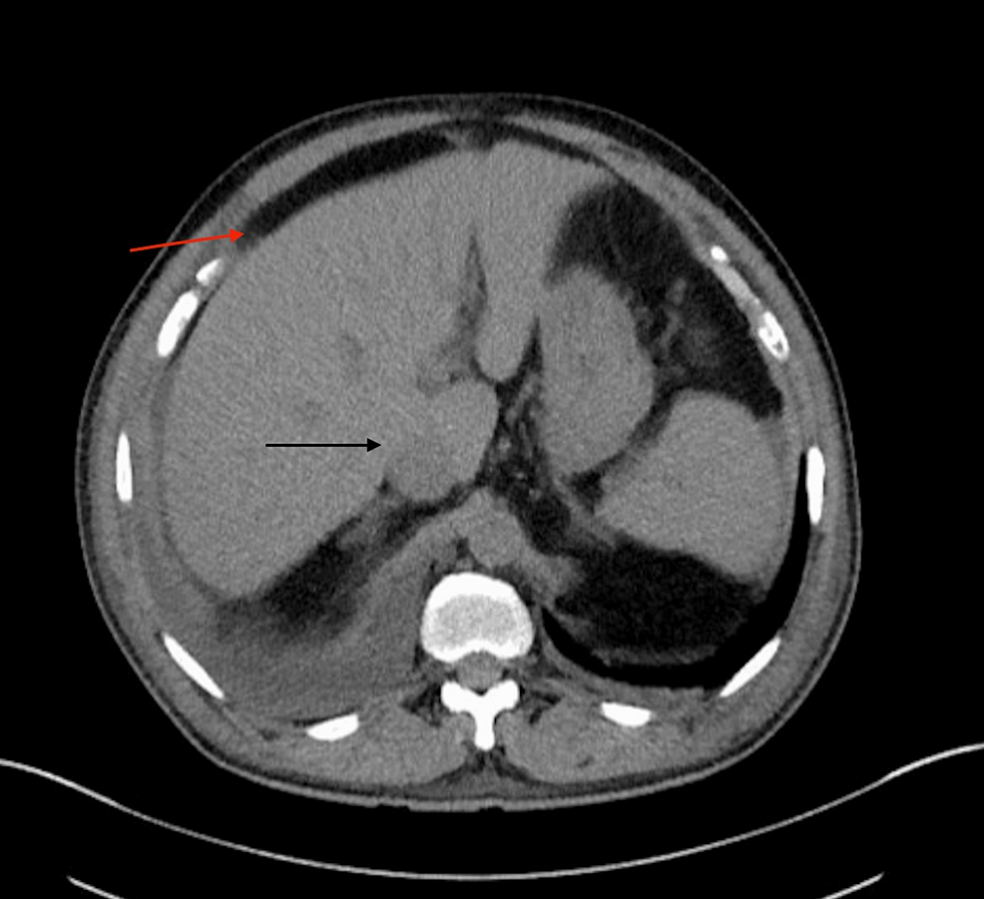
Computed tomography of the abdomen showing ascites (red arrow) and pericholecystic edema (black arrow)

**Figure 2:**
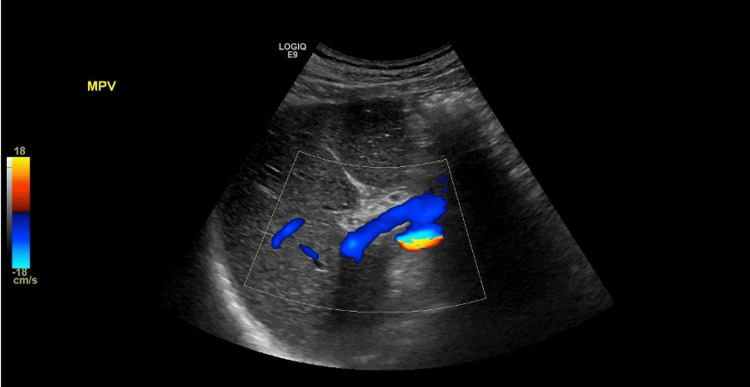
Ultrasound Doppler showing portal vein thrombosis

Laboratories were unremarkable for leukocytosis, thrombocytopenia, abnormal coagulation studies, electrolyte abnormalities, negative smooth muscle antibodies, and mitochondrial antibodies. D-dimer was not obtained in the initial presentation. Computed tomography (CT) of the abdomen and pelvis without contrast was remarkable for bilateral pleural effusions, the right being greater than the left; gallbladder thickening and pericholecystic edema; thickening and edema in the region of descending duodenum wall; and abdominal ascites (Figure 1). Abdomen ultrasound (US) showed normal liver echogenicity, mild gallbladder wall thickening, and a trace of ascites. Peritoneal fluid analysis showed a serum ascites albumin gradient (SAAG) of 1.3 g/dL and ascitic protein of 2.9 g/dL, suggesting heart failure, sinusoidal obstruction syndrome, or early Budd-Chiari syndrome. A transthoracic echocardiogram study showed mild diastolic dysfunction with preserved left ventricular systolic function (ejection fraction, 50-55%). Since acute heart failure was not the cause of the ascites, a liver US duplex was ordered, which showed PVT (Figure 2). Factor V Leiden mutation, protein C and protein S deficiency, and antithrombin mutation testing were negative. Both ascites and renal function improved with treatment with apixaban. The patient was discharged on oral anticoagulation therapy with apixaban. After six months of therapy, a venous duplex of the abdomen was repeated, which demonstrated the resolution of the thrombus. Colonoscopy and endoscopy were unremarkable for malignancy or findings suggestive of portal hypertension.

## Discussion

The most common symptoms among non-malignant and non-cirrhotic acute PVT cases are abdominal pain (up to 91%), followed by fever (53%) and ascites (38%) [5], similar to the presenting symptoms of our case. Initial testing included CT of the abdomen without contrast since contrast administration was contraindicated, given his acute kidney injury. US duplex of the liver is the modality of choice when a CT abdomen and pelvis with contrast could not be obtained, and it has been shown that US has a reported sensitivity of 89-93% and a specificity of 92-99% [6,7]. The case above demonstrated a rare presentation of PVT with ascites precipitated after two weeks from an MVA. This patient without a prior history of coagulopathy, malignancy, or family history of coagulable disorders, as described in inherited PVT, is considered a less likely etiology in this case. During his initial presentation, a d-dimer and fibrin were not ordered as the patient’s presentation was unlikely to be secondary to PVT. PVT is common in patients who already have liver cirrhosis diagnosis [1,2], which was excluded upon clinical workup. Among non-cirrhotic patients, local risk factors are known to account for the development of acute PVT due to direct injury to the portal vein during surgical interventions or indirect injury secondary to intra-abdominal inflammation process, including pancreatitis, appendicitis, cholecystitis, peritonitis, and among other etiologies, which were excluded as well [8]. Thus, in this case, the primary risk factor that precipitated the development of ascites was the traumatic MVA that the patient experienced a few weeks prior. Most case reports regarding acute PVT that are associated with abdominal trauma are rare. They have demonstrated the life-threatening nature of the diagnosis and highly morbid short-term complications, including bowel ischemia, septic thrombosis, and multi-organ failure, and long-term complications, such as portal hypertension [9,10]. Our case shows the importance of pursuing the correct diagnosis, and this patient could have had worse complications if PVT was not diagnosed early. Due to improved diagnostic techniques, early recognition, and prompt anticoagulant treatment initiation, a five-year survival rate in cases of acute non-cirrhotic PVT has improved by up to 85% [10].

## Conclusions

PVT is a rare occurrence in a rather healthy person without risk factors. Oftentimes, symptoms are broad and non-specific, which makes its diagnosis even more challenging. This case depicts a rare presentation of ascites in a non-cirrhotic patient with acute PVT in the setting of blunt abdominal trauma without acute complications. We aim to highlight the importance of accurate diagnosis, treatment, and management of atypical acute cases, such as our patient in a multi-disciplinary approach that will decrease the risk of long-term complications, improve survival rate, and optimize their quality of life.

## References

[1] Harmanci O, Bayraktar Y (2007). Portal hypertension due to portal venous thrombosis: etiology, clinical outcomes. World J Gastroenterol.

[2] Primignani M (2010). Portal vein thrombosis, revisited. Dig Liver Dis.

[3] Gupta R, Mittal P, Sekhon PS, Mittal A, Kaur H, Aamir M (2017). Acute post traumatic portal venous thrombosis associated with shattered spleen: a case report. Indian J Radiol Imaging.

[4] Gopal SV, Smith I, Malka V (2009). Acute portal venous thrombosis after blunt abdominal trauma. Am J Emerg Med.

[5] Plessier A, Darwish-Murad S, Hernandez-Guerra M (2010). Acute portal vein thrombosis unrelated to cirrhosis: a prospective multicenter follow-up study. Hepatology.

[6] Tessler FN, Gehring BJ, Gomes AS, Perrella RR, Ragavendra N, Busuttil RW, Grant EG (1991). Diagnosis of portal vein thrombosis: value of color Doppler imaging. AJR Am J Roentgenol.

[7] Bach AM, Hann LE, Brown KT, Getrajdman GI, Herman SK, Fong Y, Blumgart LH (1996). Portal vein evaluation with US: comparison to angiography combined with CT arterial portography. Radiology.

[8] Haris M, Thachil J (2017). Portal vein thrombosis - a primer for the general physician. Clin Med (Lond).

[9] Handa P, Crowther M, Douketis JD (2014). Portal vein thrombosis: a clinician-oriented and practical review. Clin Appl Thromb Hemost.

[10] Samant H, Asafo-Agyei K, Garfield K (2022). Portal vein thrombosis. https://www.ncbi.nlm.nih.gov/books/NBK534157/.

